# The relationship between psychological distress and the nursing humanistic care demands in postoperative cancer inpatients: a cross-sectional study

**DOI:** 10.1186/s12912-024-01704-7

**Published:** 2024-01-09

**Authors:** Fengyan Ma, Yajing Zhu, Yan Liu

**Affiliations:** Department of Thoracic Surgery. National Cancer Center/National Clinical Research Center for Cancer/Cancer Hospital, Chinese Academy of Medical Sciences and Peking Union Medical College, Beijing, 100021 China

**Keywords:** Cancer patient, Psychological distress, Nursing humanistic care demands

## Abstract

**Purpose:**

We aimed to investigate cancer patients' experiences of psychological distress after surgery and the factors that influence it, and to analyze the relationship between this and the nursing humanistic care demands.

**Methods:**

This study used a convenience sampling method to survey 432 cancer patients undergoing surgical treatment in the specialized cancer hospital in Beijing. The survey used socio-demographic information, the Distress Management Screening Measures, and the Nursing Humanistic Care Demands questionnaire. Questionnaire Star was used to collect data online. SPSS24.0 software was used to test the relationship between psychological distress and nursing humanistic care demands.

**Results:**

The mean scores for psychological distress and nursing humanistic care demands were 3.95 ± 2.71 and 147.02 ± 19.88, respectively, and showed a moderately positive correlation. The main issues that caused psychological distress in patients were: worry, financial problems, surroundings, nervousness, sleep, and pain. Regression analysis showed that gender, financial burden, personality trait, and need for humanistic care in nursing explained 24.5% of the total variance in the model and were independent predictors of psychological distress.

**Conclusion:**

Cancer inpatients have significant psychological distress after surgery and exhibit high levels of nursing humanistic care demands. This study fills the research gap on humanistic care for psychological distress management, nursing humanistic care demands positively predicted psychological distress. Nursing staff should pay attention to the psychological suffering of patients and develop individualized care measures to alleviate their psychological suffering by accurately identifying their nursing humanistic care demands.

## Introduction

According to the latest data from the Global Cancer Statistics 2020, there are approximately 19.3 million new cancer cases and nearly 10 million cancer deaths, with the highest incidence rates for breast, lung, and colorectal cancer [1]. According to 2015 research data, the cancer incidence rate in China is 285.83/100,000, and the mortality rate is 170.05/100,000 [2]. Surgery plays a vital role in cancer treatment, but patients can exhibit different physical and psychological symptoms of distress during treatment [3]. Some studies have shown that perioperative patients suffer from varying degrees of pain, vomiting, dizziness, anxiety, depression, and fear, which diminishes their ability to cope with cancer, reduces treatment compliance, and seriously affects the recovery process and quality of life of patients [4–6]. Pain and vomiting, as common postoperative physical symptoms of distress, can lead to unpleasant psychological problems for patients [7]. The National Comprehensive Cancer Network defines psychological distress in cancer patients as a multifactorial, unpleasant emotional experience, including psychological, social, and physical, that affects the patient's ability to cope effectively with the disease [8]. Studies have shown that the prevalence of psychological distress among Chinese cancer patients is 57.0%, which is affected by sleep, pain, temperament traits, coping styles, and financial problems, and seriously affects patients' quality of life [9]. In 2015, foreign research scholars developed five steps necessary to carry out routine psychosocial distress screening. These five steps are as follows: (1) screening, (2) evaluating, (3) referring, (4) following up, and (5) documenting and quality improvement [3]. Chinese clinical healthcare professionals are increasingly emphasizing symptom management in the perioperative period for cancer patients. However, the focus is on managing physical symptoms at the expense of the psychological distress that arises from the distress of physical symptoms. Also, research on psychological distress management is still in the screening and assessment stage, and no management system has been established [10].

Some studies have shown that cancer patients' unmet care demands predict their psychological distress points [11–13]. Demands are a human instinct, a state of dependence on the conditions for survival and development [14]. Humanistic care nursing based on Watson's care theory means that nurses pay attention to the demands of patients, respect their lives and health, and provide patients with the necessary medical and technical services as well as spiritual, cultural, and emotional services to meet the physical and other services to meet their physical and mental health demands [15]. Studies have shown that implementing humanistic care for cancer patients can improve their pain, psychological state, and ability to cope with their illness [16–18]. However, there is a discrepancy between nurses' perceptions of patients' humanistic care demands and patients' actual expectations [19]. This situation leads to the inconsistent transmission of information about humanistic care demands between nurses and patients, limiting the effectiveness of humanistic care in nursing practice. Therefore, healthcare staff should accurately identify patients' nursing humanistic care demands before implementing humanistic care.

Based on needs theory and humanistic care theory, we hypothesize that nursing humanistic care demands influence psychological distress in cancer surgery patients. Healthcare professionals should first identify the humanistic care demands of patients when caring for patients undergoing cancer surgery. By developing individualized humanistic care measures to alleviate patients' unpleasant symptoms of worry, sleep, and pain associated with surgical treatment. We aim to investigate the relationship between psychological distress and the nursing humanistic care demands in postoperative cancer inpatients to relieve patients' psychological distress and improve their ability to cope with their illnesses.

## Methods

### Study design

A cross-sectional design was used.

### Participants and settings

A convenience sampling method was used. A total of 432 patients with cancer who were hospitalized for surgery in a specialized cancer hospital in Beijing from October 2021 to October 2022 were selected. We recruited patients who underwent lung, nerve, urinary, head and neck, pancreas stomach, bone hepatobiliary, mammary gland, esophagus, female reproduction, and colorectal. Informed consent was retrieved from each patient. This survey addresses the feelings of patients in the early postoperative period when patients are more physically and energetically able to cooperate with the survey on the first postoperative day compared to the day of surgery. On the first postoperative day, the investigator instructed patients to scan the QR code generated by the online questionnaire star on their mobile phones and to fill in the questionnaire. Inclusion criteria were as follows: (i) the cancer patients who underwent surgery; (ii) the patients are aware of their condition, and (iii) informed consent and voluntary participation in this study. Exclusion criteria were as follows: (i) language and communication impairments; hearing impairments, and cognitive impairments; (ii) uncooperative patients; (iii) Patients with severe combined damage to other vital organs (such as liver and kidney), and (IV) Patients with psychiatric disorders.

The sample size follows the sampling formula of the rate $n=\frac{{\bigcup }_{a/2}^{2}\pi \left(1-\pi \right)}{{\delta }^{2}} ,\uppi = 0.3,\mathrm{ \delta }=0.05, n=\frac{1.96*1.96*0.3*(1-0.3)}{0.05*0.05}, n=323$, and considering a 20% of the invalid questionnaires, *n* = 388. The calculated sample size was at least 388, and the final sample size in this study was 432.

### Survey tools

Patient socio-demographic information, including age, gender, education, place of residence, payment method, monthly family income, marital status, children's status, whether living alone, number of episodes, family relationship, financial burden, religious belief, any co-morbidities, personality trait, and cancer site. Of these, financial burden and personality traits were reported by patients after self-assessment.

Distress Management Screening Measure (DSSM) consists of Distress Thermometer (DT) and Problem List (PL). The DT was developed by Roth et al. [20] as a single-item psychological distress self-assessment tool. The DT consists of an 11-point self-report scale ranging from "no distress" (0) to "very distressed" (10). Patients are led to rate their average level of psychological stress in the last week (higher scores indicate greater psychological stress). The NCCN considers DT ≥ 4 clinically.

significant psychological distress [21]. The PL recommended by NCCN contains a list of 36 possible problems categorized into five domains (practical, family, emotional, physical, and religious concerns). However, the Chinese version of PL contains 40 references. Patients were asked to describe their problems of the past week by answering 'yes' or 'no' to each question. The NCCN recommends using DT and PL to improve referral guidance [8]. Tang [22] measured its sensitivity of 0.80 and specificity of 0.70, with good reliability and validity. The Cronbach alpha coefficient for this scale in this study was 0.841.

The Nursing Humanistic Care Demands Scale was developed by Li et al*.* [23] in 2017 to assess patients' nursing humanistic care demands. The scale was developed based on patient self-report and humanistic care theory. The scale consists of 35 items across six dimensions, including humanitarianism, professional behavior of nurses, participation in decision-making, learning and coping, inpatient environment,

psychological and social support. Likert five-point scale was used (1 = strongly disagree, 2 = disagree, 3 = general, 4 = agree, 5 = strongly agree). Scores on the scale ranged from 35 to 175, with higher scores representing higher nursing humanistic care demands. The total Cronbach alpha coefficient of the scale is 0.892, and the Cronbach alpha coefficient of each dimension is 0.747–0.914, with good reliability. The Cronbach alpha coefficient for this scale in this study was 0.981. The Cronbach alpha coefficient of each of these dimensions ranged from 0.837 to 0.962.

### Investigation methods

After uniform training, the researcher and two investigators formed a survey team to collect data through the face-to-face distribution of electronic questionnaires with QR codes. Verbal or written informed consent was obtained from all participants before conducting the formal survey. The questionnaires were completed by the patients themselves and their companions according to the patient's actual circumstances, using standardized guidelines to assist those who had difficulty in completing the forms. The questionnaire was checked and supplemented immediately before submission in case of omissions. In this study, 450 questionnaires were distributed, and 432 effective questionnaires were retrieved, giving an effective rate of 96.0%.

### Ethical considerations

In this study, all methods were performed by the relevant guidelines and regulations. The study was approved by the Ethics Committee of the National Cancer Center/Cancer Hospital, Chinese Academy of Medical Sciences, and Peking Union Medical College(22/055–3256). All procedures were by the ethical standards of the responsible committee on human experimentation and with the Helsinki Declaration. The data was collected after informed consent and written consent were obtained from each participant.

### Data analysis

For data cleaning, statistical description, and analysis, SPSS software (version 24.0) was used. All of the data presented in this study were normally distributed. Measurement data were expressed as mean ± SD, and count data were expressed as frequencies and percentages. Psychological distress scores were compared across socio-demographic information using t-tests and ANOVA. Pearson correlation analysis was used to compare the relationship between psychological distress and nursing humanistic care demands. Variables with statistically significant differences in the results of univariate analyses and nursing human care demands were included in the multiple regression equations for further analyses. The level of detection was taken as α = 0.05, and *P* < 0.05 indicated that the differences were statistically significant. Before the regression analyses, it was confirmed whether the data met the basic assumptions of the regression, including normal distribution of residuals, linearity, chi-squareness of variance, and multicollinearity.

## Results

### Descriptive statistics of socio-demographic information

A total of 432 cancer surgery patients were included in this study, 201 (46.5%) were male, 231 (53.5%) were female, and 89.1% were married. 50.5% of the patients were aged 46–65 years old, and most of them lived in the city (66.2%) and had university or higher education (49.1%). 72.2% of the patients were paid by medical insurance, and most have a monthly income of 1000 k ~ 5000 k. Eleven types of patients suffering from cancers of different organs were included in this study, including 75 cases (17.4%) of lung, 3 cases (0.7%) of nerve, 72 cases (16.7%) of urinary, 61 cases (14.1%) of head and neck, 44 cases (10.2%) of pancreas stomach, 8 cases (1.9%) of bone, 36 cases (8.3%) of hepatobiliary, 69 cases (16.0%) of mammary gland, 27 cases (6.3%) of esophagus, 7 cases (1.6%) of female reproduction, 30 cases (6.9%) of colorectal (Table 1).

**Table 1:** General information on cancer surgery patients (*n* = 432)

Variables	Categories	n (%) or mean ± SD	t or F value	p
Age (years)			1.336	0.264
	18 ~	139 (32.2)		
	46 ~ 65	218 (50.5)		
	66 ~	75 (17.4)		
Gender			-2.456	**0.014**
	Male	201 (46.5)		
	Female	231 (53.5)		
Education			0.082	0.921
	College or above	212 (49.1)		
	High School and Post-Secondary	97 (22.5)		
	Primary and junior middle school	123 (28.5)		
Place of residence			0.966	0.472
	Town	286 (66.2)		
	Township	109 (25.2)		
	Rural	37 (8.6)		
Payment method			1.493	0.139
	At one's own expense	34 (7.9)		
	Free medical care	25 (5.8)		
	Medical insurance	312 (72.2)		
	New Agricultural	61 (14.1)		
	Cooperative			
Monthly family income(yuan)			0.855	0.576
	< 1000	23 (5.3)		
	1000 ~ 5000	242 (56.0)		
	5001 ~ 10000	96 (22.2)		
	10000 and above	71 (16.4)		
Marital status			1.336	0.209
	Unmarried	28 (6.5)		
	Married	385 (89.1)		
	Divorced	9 (2.1)		
	Widowed	10 (2.3)		
Children's status			-0.237	0.813
	No	43 (10.0)		
	Yes	389 (90.0)		
Whether living alone			0.866	0.056
	No	368 (85.2)		
	Yes	64 (14.8)		
Incidence of disease				
	First	395 (91.4)	0.082	0.935
	Recurrence	37 (8.6)		
Family relationship			1.524	0.128
	Very poor	4 (0.9)		
	Fair	68 (15.7)		
	Very good	360 (83.3)		
Financial burden			2.571	**0.005**
	Very light	34 (7.9)		
	Commonly	245 (56.7)		
	Very heavy	153 (35.4)		
Religious			1.883	0.060
	No	414 (95.8)		
	Yes	18 (4.2)		
Any co-morbidities			1.436	0.152
	No	278 (64.4)		
	Yes	154 (35.6)		
Personality trait			-4.932	** < 0.001**
	Optimism	254 (58.8)		
	Pessimism	178 (41.2)		
Cancer site			1.18	0.302
	Lung	75 (17.4)		
	Nerve	3 (0.7)		
	Urinary	72 (16.7)		
	Head and Neck	61 (14.1)		
	Pancreas Stomach	44 (10.2)		
	Bone	8 (1.9)		
	Hepatobiliary	36 (8.3)		
	Mammary Gland	69 (16.0)		
	Esophagus	27 (6.3)		
	Female Reproduction	7 (1.6)		
	Colorectal	30 (6.9)		

*n* number of cases; mean: sample mean, *SD* Standard deviation, *t /F value* t-test/ANOVA test statistic t /F value (t statistic is presented when comparison is between two groups, while F statistic is presented when comparison is among more than two groups), *P* Probability

### General characteristics of psychological distress and nursing humanistic care demands

The psychological distress score of 432 cancer surgery patients was (3.95 ± 2.71), of which 212 had significant psychological distress (DT ≥ 4) with a prevalence of 49.1%. The prevalence of psychological distress is at an intermediate level. The main issues in the problem list which caused psychological distress were: worry in 226 cases (52.3%), financial problems in 176 cases (40.7%), and surroundings in 176 cases (40.7%), nervousness in 175 cases (40.5%), sleep in 142 cases (32.9%), and pain in 129 cases (29.9%) (Table 2). Independent t-tests and ANOVA showed that gender, financial burden, and personality traits influenced the psychological distress of the patients (Table 1).

**Table 2:** Problem list of cancer surgery patients (*n* = 432)

Variables	Categories	n (%)
**Physical Concerns**		
	External appearance	63 (14.6)
	Bathe/Dress	45 (10.4)
	Respiration	54 (12.5)
	Urinary changes	47 (10.9)
	Constipation	63 (14.6)
	Diarrhea	34 (7.9)
	Eat	99 (22.9)
	Fatigue	116 (26.9)
	Edema	36 (8.3)
	Fever	29 (6.7)
	Dizziness	15 (3.5)
	Indigestion	52 (11.8)
	Pain in the mouth	26 (6.0)
	Nausea	75 (17.4)
	Nasal dryness/ congestion	38 (8.8)
	Pain	**129** (**29.9**)
	Sex	28 (6.5)
	Dry skin	65 (15.0)
	Numbness in hands/feet	42 (9.7)
	Limited physical movement	103 (23.8)
**Practical Concerns**		
	No time to care for children/elderly	91 (21.1)
	No time or energy for housework	81 (18.8)
	Financial problems	**176** (**40.7**)
	Transportation	84 (19.4)
	Work/Study	86 (19.9)
	Surroundings	**176** (**40.7**)
**Emotional Concerns**		
	Depression	65 (15.0)
	Fear	103 (23.8)
	Loneliness	45 (10.4)
	Nervousness	**175 (40.5)**
	Sadness	90 (20.8)
	Worry	**226 (52.3)**
	Loss of interest in daily activities	60 (13.9)
	Sleep	**142 (32.9)**
	Memory/Concentration	64 (14.8)
**Family Concerns**		
	Getting along with children/elderly	36 (8.3)
	Getting along with your partner	58 (13.4)
	Getting along with friends and family	34 (7.9)
	Getting along with medical staff	40 (9.3)
**Religious**		
	Religious	2 (0.5)

The average score of nursing humanistic care demands was (147.02 ± 19.88), which is at a high level, and the scores of the dimensions are shown in (Table 3).

**Table 3:** Nursing humanistic care demands scores of cancer surgery patients (*n* = 432)

**Variables**	Total score	Average score by item
Humanitarianism	25.12 ± 3.98	4.19 ± 0.67
Professional behavior of nurses	50.61 ± 6.97	4.22 ± 0.58
Participation in decision-making	16.89 ± 2.45	4.23 ± 0.62
Learning and coping	21.21 ± 3.09	4.25 ± 0.62
Inpatient environment	16.58 ± 2.64	4.15 ± 0.66
Psychological and social support	16.61 ± 2.69	4.16 ± 0.67
**Nursing humanistic care demands scores**	147.02 ± 19.88	4.21 ± 0.57

### Analysis of nursing humanistic care demands and correlation with psychological distress in cancer patients

The total psychological distress score was positively correlated with the total nursing humanistic care demands score (*r* = 0.418, *P* < 0.001) and positively correlated with all dimensions(*r* = 0.355 to* r* = 0.402, *P* < 0.001). It indicates that the higher the nursing humanistic care demands score of cancer surgery patients, the more significant their psychological distress level (Table 4).

**Table 4:** Analysis of nursing humanistic care demands and correlation with psychological distress

Variables	1	2	3	4	5	6	7	8
	*r*(*P*)	*r*(*P*)	*r*(*P*)	*r*(*P*)	*r*(*P*)	*r*(*P*)	*r*(*P*)	*r*(*P*)
1. Psychological distress	1	-	-	-	-	-	-	-
2. Humanitarianism	0.369**	1	-	-	-	-	-	-
3. Inpatient environment	0.393**	0.816**	1	-	-	-	-	-
4. Professional behavior of nurses	0.390**	0.747**	0.811**	1	-	-	-	-
5. Learning and coping	0.368**	0.646**	0.734**	0.898**	1	-	-	-
6. Participation in decision-making	0.355**	0.659**	0.711**	0.861**	0.885**	1	-	-
7. Psychological and social support	0.402**	0.699**	0.753**	0.834**	0.805**	0.832**	1	-
8. Nursing humanistic care demands	0.418**	0.847**	0.884**	0.966**	0.915**	0.901**	0.895**	1

*r* Correlation coefficient

*P* Probability

^**^P < 0.001

### Regression analysis of psychological distress

Multiple regression analyses were conducted with psychological distress score as dependent variable, significant variables in univariate analysis of variance (gender, financial burden, and personality trait) and nursing humanistic care demands as independent variables. The independent variables were coded as follows: gender, male = 1, female = 2; financial burden, very light = 1, commonly = 2, very heavy = 3; personality traits, optimism = 1, pessimism = 2; humanistic care demands are substituted with actual scores. In the regression model, gender, financial burden, personality trait, and nursing humanistic care demands explained 24.5% of the total variance in the model and were independent predictors of psychological distress (Table 5).

**Table 5:** Multiple regression analysis of factors influencing psychological distress in cancer surgery patients (*n* = 432)

Model	B	SE	Beta	t	p
Constant	-7.721	1.025	-	-7.535	< 0.001
Gender	0.621	0.231	0.114	2.687	0.007
Financial burden	0.619	0.194	0.136	3.194	0.002
Personality type	1.065	0.233	0.193	4.57	< 0.001
Nursing humanistic care demands	0.053	0.006	0.389	9.192	< 0.001

F = 34.593

R^2^ = 0.245

Adjusted R^2^ = 0.238

*p* < 0.001

## Discussion

This study examined the relationship between postoperative psychological distress and nursing humanistic care demands in hospitalized cancer patients. This study confirms our hypothesis that the nursing humanistic care demands affect psychological distress in patients undergoing cancer surgery.

The psychological distress score of the patients in this study was (3.95 ± 2.71), with a prevalence rate of 49.1%, suggesting that Chinese cancer patients have significant psychological distress after surgery. The results of this study were higher than that of the findings of Zhang [24] and Zabora [25] in cancer patients (24.2%-35.1%), it may be related to the collection of data on the first postoperative day. Surgery can cause cancer patients to experience unpleasant symptoms such as pain, fatigue, anxiety, and fear, which can alter the patient's psychological state and result in high psychological distress [26]. Psychological distress can affect the patient's recovery process and compliance with treatment [27], so clinical staff should pay attention to the psychological distress of postoperative patients and can apply tools to identify the level of psychological distress effectively.

The main problems that cause psychological distress are worry, financial issues, surroundings, nervousness, sleep, and pain. Fear has become a major psychological problem in China for cancer patients after surgery. Cancer patients hospitalized for surgery are generally in the early stages of the disease with greater expectations of prognosis, making the fear of recurrence and stress more apparent [28]. The costs incurred for surgical treatment create tremendous psychological stress for patients, especially those undergoing neoadjuvant therapy, which can incur higher charges and put patients under financial pressure [29]. A study of adolescent and young cancer patients in Asia showed that the main issues that caused patients psychological distress were worry, financial problems, and treatment decisions [30]. The surgical treatment causes pain in patients in the perioperative period, even though health care takes pain very seriously and multimodal analgesic measures are used. The psychological changes of discomfort arise during the patient's transition from the social environment of home and work to the inpatient setting. The above pain and the change in surroundings become the main problems that induce psychological distress in postoperative patients, seriously affecting their social environment and quality of life [24, 31]. Therefore, to improve their compliance and prognosis, clinical staff should pay more attention to the main issues that lead to psychological distress in cancer patients after surgery.

The score of the nursing humanistic care demands in this study was (147.02 ± 19.88), which was a high level comparable to that of schizophrenic patients [32]. It shows that the humanistic care measures currently implemented in hospitals do not fully meet the actual demands of postoperative cancer patients [33]. Postoperative cancer patients in this study were highly demanded to learn surgical-related expertise and participate in clinical treatment decisions [34]. It may be because most of the patients in this study had high education levels and enhanced knowledge and mastery of surgical expertise, and thus were more eager to acquire more expertise and participate in treatment decision-making. However, clinical measures related to the dissemination of expertise and inviting patients to participate in decision-making could not meet the patients' demands. Therefore, medical staff should pay attention to the humanistic care demands of cancer patients, change traditional attitudes, provide patients with knowledge about the disease in multiple ways, improve their ability to cope with it, and enable them to participate in clinical decision-making. However, some studies have shown that nursing humanistic care is problematic in China, related of weak concepts of nurses' humanistic care, lack of competence, and scarcity of professional staff [35]. Nursing schools and clinical nursing managers should strengthen the nursing staff's awareness of humanistic care through standardized nursing humanistic care education and training and improve the ability to provide personalized and standardized nursing care. At the same time, the state should focus on training nursing talents and arranging nursing work rationally so that nurses have more time to focus on patient’s psychological problems and provide quality nursing services.

This study showed that nursing humanistic care demands had a significant positive predictive effect on psychological distress. It shows that the higher the nursing humanistic care demands of cancer patients, the higher their level of psychological distress. Nursing humanistic care means paying attention to the actual demands of patients, respecting the value of patients' lives, providing patients with diagnostic and technical services, and paying attention to their spiritual, cultural, and emotional aspects to meet their physical and mental health demands in the nursing process [36]. Based on Watson's theory of care, nursing humanistic care demands are the nursing humanistic concerns patients need to meet. Accurate knowledge of patients' humanistic care demands is the basis for nursing staff implementing humanistic care measures. Several studies have shown that implementing humanistic care in clinical settings can reduce patients' psychological stress, relieve anxiety and depression, reduce the incidence of adverse emotions, improve life quality and satisfaction, and harmonize the nurse-patient relationship [17, 37, 38]. To meet the humanistic care demands of patients in nursing to reduce psychological distress caused by physical, psychological, and social problems. Therefore, medical staff can reduce patients' psychological distress by effectively identifying their humanistic demands and developing effective humanistic care measures from the patient's perspective.

Multiple regression analyses in this study showed that gender, financial burden, and personality traits predicted patients' psychological distress to varying degrees. This observation may be attributed to the fact that a significant proportion of female patients in this study were afflicted with breast or gynecological cancers, with breast reconstruction and fertility concerns compounding the psychological distress experienced by this cohort [39]. Patients undergoing high financial burdens exhibit elevated levels of psychological distress, which aligns with the finding that financial difficulties are the primary cause of psychological distress among patients in China, as evidenced by the survey responses table. This convergence of evidence supports the conclusion that financial burdens are the predominant factor influencing psychological distress among patients in China [9]. It has been shown that depressed patients, who are pessimistic and tend to see the negative aspects of the illness when faced with stress, are more likely to deliver higher levels of psychological distress [9]. It has also been demonstrated that pessimistic surgical patients exhibit higher pain levels and that pain predicts psychological distress in patients [40]. Therefore, healthcare workers should prioritize breast and gynecological cancer patients and thoroughly consider their social image and physical needs in treatment decisions. Reduce unnecessary tests and treatments during disease diagnosis and treatment to reduce patients' financial burden. For postoperative cancer patients, understand their personality traits, establish a pain management mechanism, and guide them to face the disease correctly to reduce their psychological distress and increase treatment compliance.

## Limitations

As this study was a cross-sectional study and limited to one hospital in a tertiary cancer hospital, there are still many shortcomings, and a multi-center large sample study could be organized in the future. This study did not include the pathological staging of the patients, was limited to understanding the patient's condition on day 1 after surgery, and included an uneven proportion of different cancer types. In future studies, more variables should be included to examine the trajectory of perioperative psychological distress in different types of cancer patients and to explore effective measures to reduce psychological distress.

## Conclusion

This study is the first to explore the correlation between postoperative psychological distress and nursing humanistic care demands in cancer patients, linking nursing humanistic care demands to the somatic, social, and psychological factors that cause postoperatively induced psychological distress in cancer patients. This study provides a direction and theoretical basis for clinical healthcare workers to develop measures to reduce patients' psychological distress. Healthcare workers should pay attention to the pain, sleep disturbance, worry, and financial burden that cancer patients experience after surgery and develop care strategies by identifying patients' unpleasant symptoms and humanistic care demands.

## Data Availability

All data generated or analyzed during this study are included in this published article.
